# Surveying Health Professions Students’ Use of Generative AI Applications

**DOI:** 10.1007/s40670-025-02429-1

**Published:** 2025-06-02

**Authors:** Emily C. Harris, Amy Baldwin, Kathy Davies, Julie K. Gaines, Janette Hill, Shafer Tharrington, Edwin V. Sperr

**Affiliations:** 1 Department of Libraries, Augusta University, Augusta, GA USA; 2 Augusta University/University of Georgia Medical Partnership, Athens, GA USA; 3 Mary Frances Early College of Education, University of Georgia, Athens, GA USA; 4 University Libraries, University of Georgia, Athens, GA USA

**Keywords:** Generative AI, Medical education, Information-seeking behavior, Health professions education

## Abstract

While there has been an explosion of interest in the use of generative AI (GenAI) applications in medical education over the past 2 years, relatively little is understood about how health professions students are using this technology. This study aimed to answer these questions by surveying students about their GenAI use. Unsurprisingly, this survey revealed that many students were indeed using these applications, most often for information retrieval.

## Background

The debut of ChatGPT in late 2022 [1] heralded a new explosion of interest in the idea of using artificial intelligence, particularly generative AI (GenAI), in medical education. There has been much speculation on what the future holds [2, 3], but less is understood about how these technologies are currently being used by health professions students. Most usage studies to date have focused on *attitudes* about GenAI or focused on non-health professions students. This study is designed to help fill this information gap by directly surveying health professions students at Augusta University about how often and why they are using GenAI applications.

## Activity

The investigators created a 34-item survey (see Appendix) on the current use of GenAI by health professions students at Augusta University, including those enrolled at the Augusta University/University of Georgia Medical Partnership campus in Athens, GA. Questions included which applications students were using, how often they used them, and the purposes for which they were used. Students were asked in which program they were enrolled, as well as basic demographic information. Survey items were loaded into Qualtrics for online administration.

The investigators obtained approval for this project from the campus Division of Institutional Effectiveness (DIE) and a formal designation as “exempt” by the Augusta University Institutional Review Board. As part of the approval process, DIE provided 1600 randomly selected email addresses for health professions students. Each of these students was sent an emailed invitation to participate, including information about the project as well as a link to the survey itself. Printed flyers with a QR-encoded link to the survey were also posted on both the Augusta and Athens campuses. Emails and reminders were sent over a 1-week period at the end of March 2024, and the flyers were posted at the same time. Responses received through the following month were analyzed.

## Results and Discussion

The investigators received a total of 103 responses, 88 of which came in response to the emailed invitation, 15 from the flyer link, and one from a link forwarded by another respondent. One hundred of these were from currently enrolled health professions students at Augusta University. This number of responses was enough to draw broad conclusions about student use of these applications, though not sufficient to make reliable distinctions between subgroup use. Survey respondents were broadly similar to those who were sent email invitations, both in terms of demographic characteristics and field of study. The biggest differences were that “White” students were somewhat more likely to respond (60% self-identified respondents vs. 53% of invitees so identified in campus records) and Allied Health students were somewhat less likely (16% of respondents vs. 23% of invitees).

It is clear that many students are currently using GenAI applications. Fully 70% of respondents reported “yes” when asked “Have you ever used a Generative AI/Large Language Model application or website?” with only 11% reporting that such use was in response to being introduced to or assigned them in class. While 9% reported using them for “mostly academic use” and 26% for “personal use only,” the majority (66%) reported a mix of academic and personal use.

We attempted to quantify how often those students who were familiar with GenAI applications were using them, as well as which applications they were using. Out of those survey responses that fit our inclusion criteria, 30% reported using them once a week or more, with 19% using them “several times a week” and 11% “once or twice a week.” A further 16% reported using GenAI applications “a couple of times a month” and 6% having “tried it a few times, but don’t currently use them.” Out of the 51 respondents to the question “Compared to when you first started using these applications, you know find yourself using them…,” 37% of respondents reported using these applications “more often” than when they started, with 22% using them less often and 41% about as often as before. By far the predominant individual application used by these respondents on a regular basis was *ChatGPT* (72%), with much smaller numbers using Google *Bard* or *Gemini* (9%), Microsoft’s *Bing AI/Copilot* (5%), or *DALL-E* (5%).

**Fig. 1:**
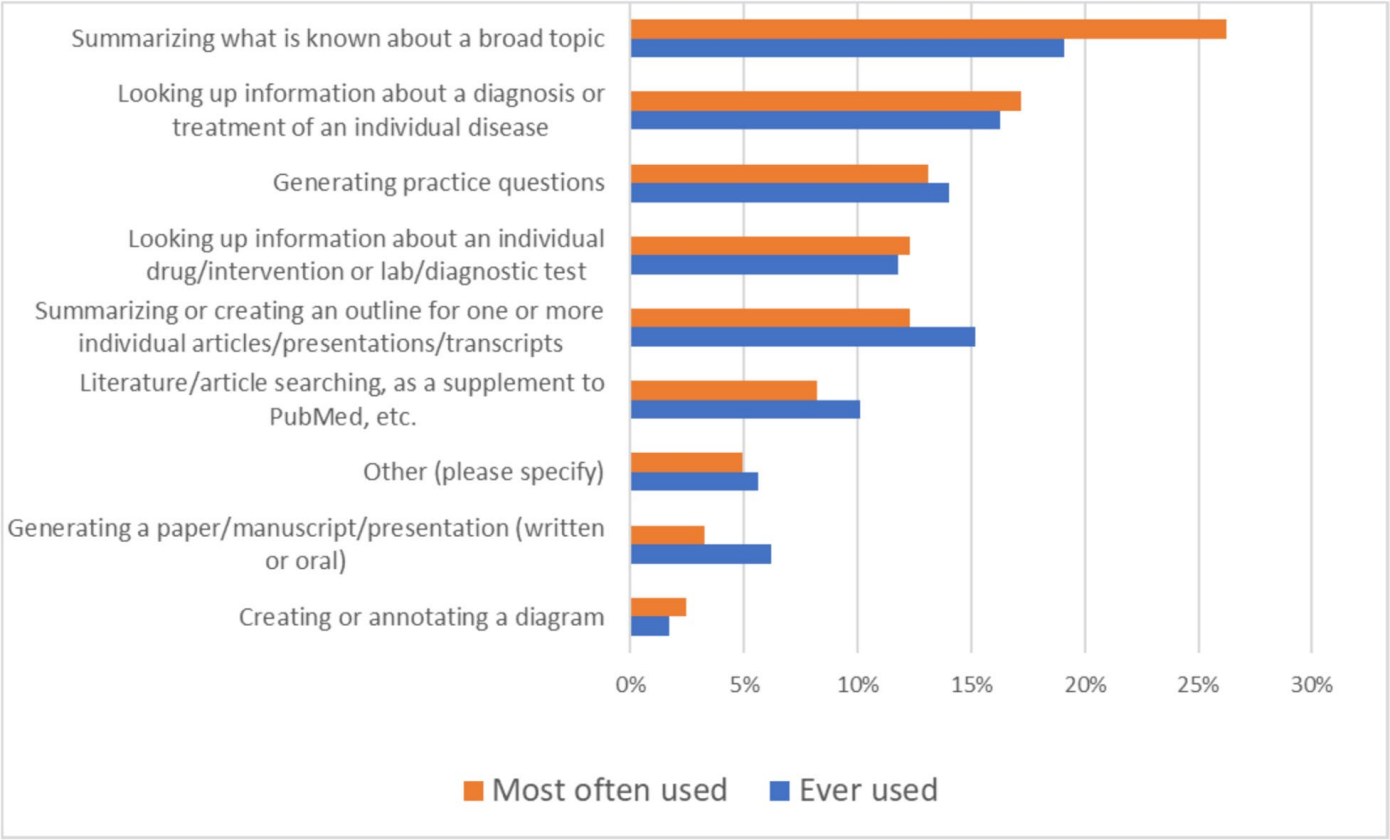
Types of tasks that respondents have ever used a GenAI application for (178 total responses) and types for which that they most often use them at the present (122 responses)

One goal of this study was to gain a more granular understanding of what kinds of *tasks* students are using GenAI applications for. We asked students to identify which types of tasks they had *ever* used these applications for as well as how they were *currently* using them. These results (summarized in Fig. 1) show that the predominant use of these tools is for information retrieval. Indeed, a full 64% of responses were for such tasks as “Summarizing what is known about a broad topic” and “Looking up information about a diagnosis or treatment of an individual disease.” While some respondents indicated that they had tried or were currently using GenAI applications for more sophisticated tasks such as generating practice questions or creating presentations, these kinds of uses were notably less common. There were few differences between types of tasks between the ever-used vs. currently using groups with the exception of “Summarizing a broad topic” which grew from 19% of responses in the former to 26% in the latter.

Given the well-documented tendency of current GenAI applications to misreport facts or invent citations [4], respondents were asked how often they encountered these confabulations or “hallucinations.” Fourteen percent of respondents (7 of 49) indicated that they “never” found such mistakes, while 33% responded they “rarely” and 35% reported they only “sometimes” encountered them. When asked about their strategies for coping with such situations, the most common response was that they would simply consult another source. This low frequency of mistake recognition by our respondents seems difficult to reconcile with the confirmed incidence of such confabulations in the medical context [5].

While much has been written about potentially transformative uses of GenAI in health professions education, respondents in our survey are most often using these applications for basic information retrieval tasks. This is striking given that medical educators and librarians devote such substantial resources to the acquisition and promotion of the textbooks, databases, and point-of-care resources designed for precisely these kinds of uses. Why would a student choose *ChatGPT* for the kind of task that is arguably better performed with *Micromedex*, *UpToDate*, or *Harrison’s?* Is their preference just a matter of ease of use, or does it come from a failure to communicate what “traditional” summative resources do well? Or is it perhaps a desire by these students to only deal with the *broadest* possible summary rather than having to integrate information from multiple resources?

Our results would indicate that approximately a third of all AU health professions students were using GenAI applications at least once a week in the Spring of 2024. However, while the demographic profiles of respondents were very similar to those of the invitees, the low response rate from email invitations (less than 6%) and relatively high proportion of responses (18%) from the convenience sample of flyer links and referrals do raise the question of response bias — is such regular use really that common, or were we just more likely to hear from students that were already enthusiastic about this technology? Therefore, it is worth noting that such a frequency of usage is broadly in line with the 21.6% of medical students using *ChatGPT* for studying on a daily or weekly basis reported by Ganjavi et al. [6] and the 43.7% using it for study weekly or more often reported by Zhang et al. [7].

Our results indicate that there is a substantial cohort of students using these technologies to perform information retrieval tasks on a regular basis. Further quantifying the size of this group as well as qualifying exactly *why* they are using GenAI applications for such tasks is beyond the scope of this study, but such answers hold substantial implications for both library collection development and health professions pedagogy. It is particularly concerning that many students are using these GenAI tools for fact-finding purposes without understanding how unreliable these tools are for that function.

## Data Availability

While detailed individual survey responses are unavailable due to privacy concerns, aggregate data are available at the project site: https://osf.io/a6e4v/.

## Appendix

### Supporting Materials

A more detailed readout of our data and other supporting files can be found at the project site: https://osf.io/a6e4v/.

### Survey Instrument

Are you an Augusta University or Medical Partnership health professions (Medical, Dental, Nursing, Allied Health, or other) student?

- Yes
- No

Which age range are you in?

- Under 18
- 18—22
- 23—27
- 28—32
- 33—37
- 38—42
- 43—50
- 51—60
- 61 or older

How do you identify?

- Man
- Woman
- Non-Binary
- Other/Prefer not to say

Which race or ethnicity best describes you?

- American Indian or Alaskan Native
- Asian/Pacific Islander
- Black or African American
- Hispanic
- White
- Mulitple ethnicities/Other

What type of student are you?

- Allied Health (please specify program)
- Medical
- Nursing (please specify program)
- Dental
- Other (Please specify program)

On which campus do you attend classes?

- Augusta University in Augusta, GA
- Medical Partnership in Athens, GA
- College of Nursing in Athens, GA
- Other/Satellite Campus (please specify)

Have you ever used a Generative AI/Large Language Model application or website?

- Yes
- No

Were you introduced to these applications (or assigned to use one of them) as part of a class in your current program?

- Yes
- No

What have you used these applications for?

- Personal use only
- Mostly academic use
- Mixture of personal and academic use

How often do you use these applications?

- Tried it a few times, but don’t currently use them
- A couple of times a month
- Once or twice a week
- Several times a week

Which of these applications have you tried before? Check all that apply.

- ChatGPT
- Google Bard/Gemini
- Microsoft Bing AI/Copilot
- Midjourney
- Claude
- DALL-E
- Stable Diffusion
- Adobe Firefly
- Other (please specify)

Which applications do you *currently* use, if any? Check all that apply.

- ChatGPT
- Google Bard/Gemini
- Microsoft Bing AI/Copilot
- Midjourney
- Claude
- DALL-E
- Stable Diffusion
- Adobe Firefly
- Other (please specify)

Compared to when you *first* started using these applications, you *now* find yourself using them…

- Less often
- More often
- About as often as before

Which academic tasks have you ever used these applications for? Check all that apply.

- Creating or annotating a diagram
- Generating practice questions
- Summarizing or creating an outline for one or more individual articles/presentations/transcripts
- Summarizing what is known about a broad topic
- Generating a paper/manuscript/presentation (written or oral)
- Looking up information about a diagnosis or treatment of an individual disease
- Looking up information about an individual drug/intervention or lab/diagnostic test

- Literature/article searching, as a supplement to PubMed, etc.
- Other (please specify)

Which academic tasks, if any, do you *most often* use these applications for now? Check all that apply.

- Creating or annotating a diagram
- Generating practice questions
- Summarizing or creating an outline for one or more individual articles/presentations/transcripts
- Summarizing what Is known about a broad topic
- Generating a paper/manuscript/presentation (written or oral)
- Looking up information about a diagnosis or treatment of an individual disease
- Looking up information about an individual drug/intervention or lab/diagnostic test
- Literature/article searching, as a supplement to PubMed, etc.
- Other (please specify)

Have you ever encountered a mistake/incorrect information (aka,”a hallucination”) when using one of these applications for academic tasks?

- No, never
- Yes, but very rarely
- Yes, sometimes
- Yes, often
- Not sure

If you do encounter mistakes/wrong information when using these applications, what do you do about it?

Would you encourage other students to use these applications?

- Yes
- No

Are there any other thoughts you would like to share with us about how you use these applications?
